# Binding partner- and force-promoted changes in αE-catenin conformation probed by native cysteine labeling

**DOI:** 10.1038/s41598-019-51816-3

**Published:** 2019-10-25

**Authors:** Ksenia Terekhova, Sabine Pokutta, Yee S. Kee, Jing Li, Emad Tajkhorshid, Gerald Fuller, Alexander R. Dunn, William I. Weis

**Affiliations:** 1 Departments of Structural Biology and Molecular & Cellular Physiology, Stanford University School of Medicine, Stanford, CA 94305 USA; 2 Department of Chemical Engineering, Stanford University, Stanford, CA USA; 3 Departments of Chemistry, Chemical and Biomolecular Engineering, and Center for Biophysics and Quantitative Biology, University of Illinois, Urbana, IL USA; 4 Cardiovascular Institute, Stanford University School of Medicine, Stanford, CA USA; 5 Present Address: Genentech, Inc., 1 DNA Way, South San Francisco, CA 94080 (Y.S.K.); Department of Biochemistry and Molecular Biology, University of Chicago, Chicago, IL 60637 (J.L.) USA

**Keywords:** Biochemistry, Biophysics, Structural biology

## Abstract

Adherens Junctions (AJs) are cell-cell adhesion complexes that sense and propagate mechanical forces by coupling cadherins to the actin cytoskeleton via β-catenin and the F-actin binding protein αE-catenin. When subjected to mechanical force, the cadherin•catenin complex can tightly link to F-actin through αE-catenin, and also recruits the F-actin-binding protein vinculin. In this study, labeling of native cysteines combined with mass spectrometry revealed conformational changes in αE-catenin upon binding to the E-cadherin•β-catenin complex, vinculin and F-actin. A method to apply physiologically meaningful forces in solution revealed force-induced conformational changes in αE-catenin when bound to F-actin. Comparisons of wild-type αE-catenin and a mutant with enhanced vinculin affinity using cysteine labeling and isothermal titration calorimetry provide evidence for allosteric coupling of the N-terminal β-catenin-binding and the middle (M) vinculin-binding domain of αE-catenin. Cysteine labeling also revealed possible crosstalk between the actin-binding domain and the rest of the protein. The data provide insight into how binding partners and mechanical stress can regulate the conformation of full-length αE-catenin, and identify the M domain as a key transmitter of conformational changes.

## Introduction

**Figure 1:**
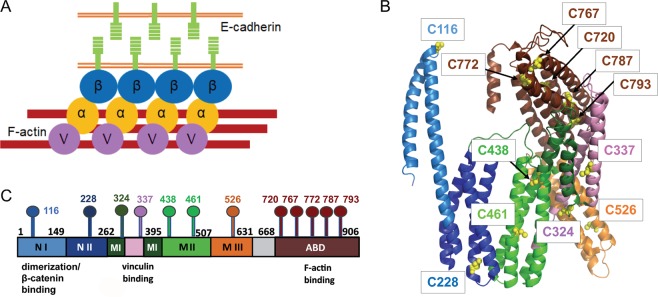
αE-catenin structure. (**A**) In AJs, E-cadherin mediates cell-cell adhesion and associates with αE-catenin (α) through β-catenin (β). Under force, αE-catenin (bound to the E-cadherin•β-catenin complex) binds to actin and other actin-binding proteins, including vinculin (V), to strengthen the AJs. (**B**) A crystal structure of αE-catenin (PDB 4IGG). The N domain, which mediates β-catenin binding and homodimerization, comprises two helical subdomains, N_I_ (light blue) and N_II_ (dark blue). The middle (M) domain comprises three 4 helix bundles, M_I_ (dark green), M_II_ (light green) and M_III_ (orange); vinculin binds to the two central helices in M_I_ (shown in pink) The F-actin binding domain (ABD) is shown in brown; the position of the ABD differs in the two copies in PDB 4IGG^26^, and chain B is shown here. The cysteine residues are shown as yellow spheres. (**C**) Primary structure of αE-catenin. The positions of cysteine residues (“lollipop” symbols) are indicated.

Adherens Junctions (AJs) are key cell-cell adhesion complexes that regulate tissue morphogenesis, differentiation and wound healing, and their disruption is associated with cancer metastasis^1–10^. In these assemblies, the extracellular portion of classical cadherins mediates homophilic cell-cell adhesion. In epithelial tissues, the cadherin intracellular domain binds to β-catenin, which in turn associates with the F-actin-binding protein αE-catenin (Fig. 1A)^11^. Notably, binding to the cadherin•β-catenin complex weakens the affinity of αE-catenin for F-actin in solution, but the binding of the cadherin•β-catenin•αE-catenin complex to F-actin is strengthened by mechanical tension^12–15^. Specifically, αE-catenin functions as a force-sensitive structural switch that displays two-state catch bond behavior: it transitions from weak to strong actin-binding states between low (<2 pN) and moderate (8 pN) forces^12^. Furthermore, at forces of approximately 5 pN, αE-catenin recruits vinculin, which is thought to reinforce the AJ through its own actin-binding activity^5,16,17^ (Fig. 1A). This functional activation is thought to be achieved by structural rearrangements in αE-catenin^18–20^. Hence, αE-catenin is the central force-sensing element of the AJ^6,11,12,16,18,19,21–25^. A challenge is to understand what conformational changes promoted by force and binding partners underlie αE-catenin function.

The structure of αE-catenin comprises a series of α-helical bundles organized into three main regions: an N-terminal (N) domain that consists of two four-helix bundles (N_I_ and N_II_) that both binds β-catenin and mediates homodimerization; a middle (M) domain consisting of three four-helix bundles (M_I_, M_II_, and M_III_) that harbor binding sites for vinculin and other protein partners; and a C-terminal, five-helix bundle termed the actin-binding domain (ABD)^18,26^ (Fig. 1B,C). Structural studies using minimal interacting domains have revealed that β-catenin binding stabilizes a conformation in which N_I_ and N_II_ are bent with respect to one another relative to their positions in the unbound molecule^27,28^. Structural and biophysical analyses have shown that the M_I_ bundle is relatively unstable, and can unfurl to expose its two central helices, which bind to vinculin^20,22,29,30^. Vinculin binding is partially inhibited through a network of salt bridge interactions among M_I_, M_II_ and M_III_, where the latter domain plays a critical inhibitory role; force is thought to disrupt these interactions and thereby promote binding to vinculin^16,20,22,31,32^. Individual ablation of several of these salt bridges, notably that between R551 in M_III_ and D503 in M_II_, enhanced binding to vinculin^18,30,33^. Steered molecular dynamics (MD) simulations, combined with mutational analysis, have identified salt bridge interactions within the M domain whose dynamics are thought to be associated with regulated vinculin binding^19,33,34^.

Despite the advances outlined above, the structural responses of full-length αE-catenin to its binding partners and force are poorly understood. Little is known about F-actin binding by αE-catenin, including the molecular mechanisms by which cadherin-β-catenin inhibits, and force promotes, binding of αE-catenin to F-actin. Here, we examine the conformational dynamics and inter-domain coupling of αE-catenin associated with its binding to cadherin•β-catenin, F-actin, and vinculin by measuring changes in native cysteine accessibility in solution. We developed a novel method to directly probe the effect of mechanical force on αE-catenin by examining changes in cysteine accessibility under physiologically relevant levels of mechanical load induced by fluid shear. Combined with structural and thermodynamic data, these experiments provide direct experimental data for structural changes associated with allosteric regulation of αE-catenin.

## Results

### αE-catenin structure and dynamics in solution examined by thiol labeling

Two crystal structures of full-length or nearly full-length αE-catenin homodimer have been reported: a 6.5 Å resolution structure of full-length αE-catenin in which the ABDs were disordered (PDB 4K1N)^18^, and αE-catenin 82–906 at 3.7 Å (PDB 4IGG) in which the ABDs in each protomer were ordered, but adopted different positions with respect to the rest of the molecule^26^. Small angle x-ray scattering (SAXS) analysis of the full-length protein suggested a more extended conformation than that observed in the 4IGG structure^18,35^. The absence of the N-terminal 81 residues, and/or that the crystals were dehydrated to improve resolution of the 4IGG structure, raised the possibility that the positions of the ABDs in 4IGG do not represent the solution structure of the αE-catenin dimer. Therefore, we determined the crystal structure of the αE-catenin 82–883 dimer at 4.0 Å resolution without dehydration, and also examined its structure in solution by SAXS. The ABDs in αE-catenin 82–883 crystals were disordered, and the protein is extended in solution (Supplemental Information, Supplemental Fig. 1), demonstrating that the disorder of the ABDs and the extended nature of the molecule are not due to the absence of the first 81 residues. Whether the compact structure with ordered ABDs visualized in the 4IGG structure represents a minor population of the solution ensemble remains to be determined. Evidence presented below is consistent with communication between the ABD and the N-M region of αE-catenin.

We used site-specific thiol labeling followed by mass spectrometry to determine changes in native cysteine accessibility in isolated wild-type and R551A mutant αE-catenin, in its complexes with E-cadherin and β-catenin, F-actin and vinculin, and under force when bound to F-actin. For all experiments in solution, our strategy was to label pre-formed, saturated complexes, so that any changes in labeling between different complexes reflect conformational differences between them. Note that although these complexes are saturated at equilibrium in these experiments, we cannot exclude that labeling of a small fraction of dissociated proteins at equilibrium may occur. More significantly, for complexes of αE-catenin with F-actin immobilized on glass (see below), saturating levels of F-actin were mixed with αE-catenin at the same time as the label, so effects of the label on αE-catenin interactions with its partners cannot be excluded. However, even if Cys modifications were to alter the structure, differences in reactivity must reflect changes in structure or dynamics. Thus, direct pairwise comparisons of cysteine labeling patterns under different conditions provides a means to derive meaningful insight into differences in αE-catenin conformation under conditions that are otherwise difficult to access (*e.g*., αE-catenin under load).

We chose the fluorescent thiol-reactive probe monobromobimane (mBBr), which is roughly the size of a tryptophan side chain, in part for a possible future application to directly label αE-catenin in cells, as mBBr can cross membranes^36^. (The expense of mass spectrometry analysis restricted our experiments to the use of only one probe.) The relatively small size of mBBr allows it to probe modest changes in conformation such as small rearrangements of interdomain interfaces expected from prior structural and computer simulation studies.

**Figure 2:**
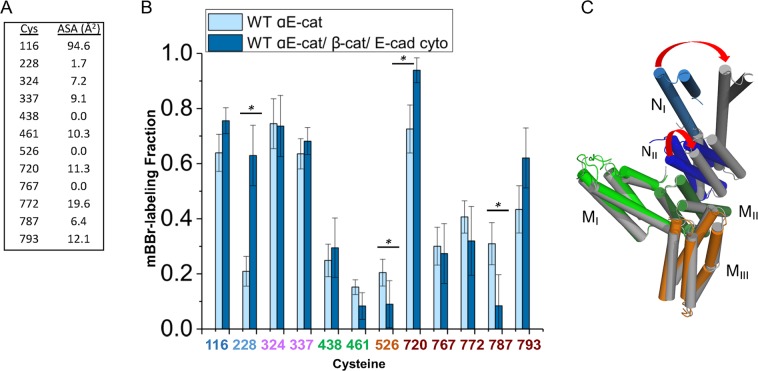
mBBr labeling of cysteines in αE-catenin. (**A**) Accessible surface area for the twelve native cysteines in αE-catenin, calculated from 4IGG chain B. (**B**) The fraction of each cysteine labeled by mBBr, as assayed by mass spectrometry, is shown for the indicated condition. Labeling of αE-catenin alone (7 technical replicates and 5 independent protein preparations) versus αE-catenin when bound to the cadherin•β-catenin complex (4 technical replicates and two independent protein preparations). p-values ≤ 0.05 are flagged with one star (*). (**C**) Superposition of protomers A and B in the crystal structure; residues 398–505 (M_II_) were aligned. Chain A is colored as in Fig. 1, and chain B is shown in grey.

Murine αE-catenin has 12 cysteine residues, which the crystal structures reveal have variable levels of solvent exposure (Fig. 2A). We established that labeling of 15 μM αE-catenin with 1.5 mM mBBr is unchanged after 20 min (Supplemental Fig. 2). Mass spectrometry revealed variable levels of mBBr incorporation at the different sites that correlated only weakly with Cys side chain exposure observed in crystal structures (Fig. 2B). This may reflect dynamics of the molecule in solution vs. the conformation observed in crystal structures. Notably, Cys 324 and Cys 337 in M_I_, which have limited solvent accessibility in crystal structures, label strongly; this region is also proteolytically sensitive^28,37^, indicating that M_I_ is relatively unstable and flexible. The mass spectrometry data were not as reliable in the ABD due to the proximity of cysteines in the digested peptides, which made determination of labeling efficiency at a given site challenging.

### Structural changes in the presence of β-catenin and F-actin

Superposition of the individual protomers in each αE-catenin dimer crystal structure shows that although the N- and M- domains have similar structures, they assume two different orientations with respect to each other due to differences in the N-M interface formed by the N_II_ and M_II_ bundles (Fig. 2C, Supplemental Fig. 3). Moreover, binding of β-catenin to the αN-catenin N domain produces a significant change in the angle between N_I_ and N_II_, which modeling suggested can alter the contact between N_II_ and M_II_ or even completely separate these two subdomains^27^. These observations suggest that the N_II_-M_II_ interface is plastic and could be a site of coupling between the N-terminal β-catenin binding domain and the rest of αE-catenin. Cysteine labeling provided evidence for this coupling: upon binding to E-cadherin•β-catenin to N_I_ (Pokutta 2014), C228 in N_II_ becomes significantly more exposed in αE-catenin (Fig. 2B). C228 packs against Y237, which is directly in the interface with M_II_ (Supplemental Fig. 3), so changes in its exposure may arise from changes in the N_II_-M_II_ interface. Significant changes in labeling were also detected at C526 (M_III_) and cysteines 720 and 787 in the ABD when WT αE-catenin is bound to E-cadherin•β-catenin. Although the available structural data do not provide an obvious explanation for these results, they do provide experimental evidence for coupling between the β-catenin binding N domain and the M and F-actin-binding domains of αE-catenin (Fig. 2B), which may underlie the inhibitory effect of E-cadherin•β-catenin binding on F-actin affinity.

**Figure 3:**
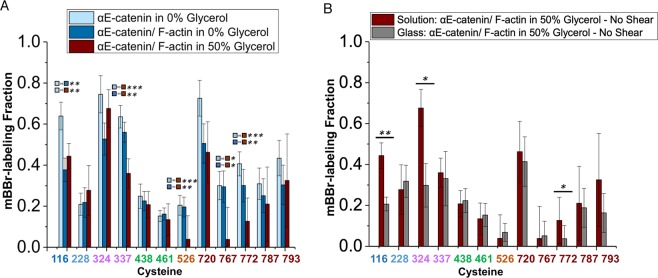
Labeling of αE-catenin in the presence of F-actin. (**A**) Labeling of αE-catenin cysteines with F-actin ± 50% glycerol. The data for αE-catenin without F-actin and glycerol are the same as those in Fig. 2B. The data for the αE-catenin/F-actin complex without glycerol are from 7 technical replicates and 4 preparations. The data for the αE-catenin/F-actin complex with 50% glycerol are from 5 technical replicates and 2 preparations. (**B**) Comparison of mBBr-labeling of αE-catenin in solution vs. immobilized on the glass surface. The data for αE-catenin on glass in the presence of F-actin and 50% glycerol are from 6 technical replicates and 6 independent protein preparations. In each figure, p-values ≤ 0.05 are flagged with one star (*), ≤0.01 with two stars (**), and ≤0.001 with three stars (***).

Relatively little is known about how the C-terminal actin-binding domain (ABD) binds to actin filaments. A recent SAXS/SANS study showed that when bound to F-actin, the αE-catenin ABD is separated from the remainder of the protein, tethered by the long loop that connects the M domain to the ABD^35^. On the other hand, binding of E-cadherin•β-catenin to αE-catenin weakens the affinity of αE-catenin for F-actin^12–15^. As noted above, association of αE-catenin with E-cadherin•β-catenin led to changes in labeling at C720 and C787 (Fig. 2B), indicating crosstalk between the ABD and the rest of the protein. Given these findings, we examined the effect of F-actin on αE-catenin cysteine labeling by mBBr. No significant differences in the ABD cysteine residues were seen ± F-actin (Fig. 3A). The one significant change occurred at C116 in the N_I_ bundle; the reduction in the labeling of this otherwise exposed residue may indicate that the ABD in a subpopulation of αE-catenin, or perhaps the F-actin filament itself, lies nearby and reduces its exposure.

### Allosteric coupling of αE-catenin domains revealed by a vinculin-binding mutant

**Figure 4:**
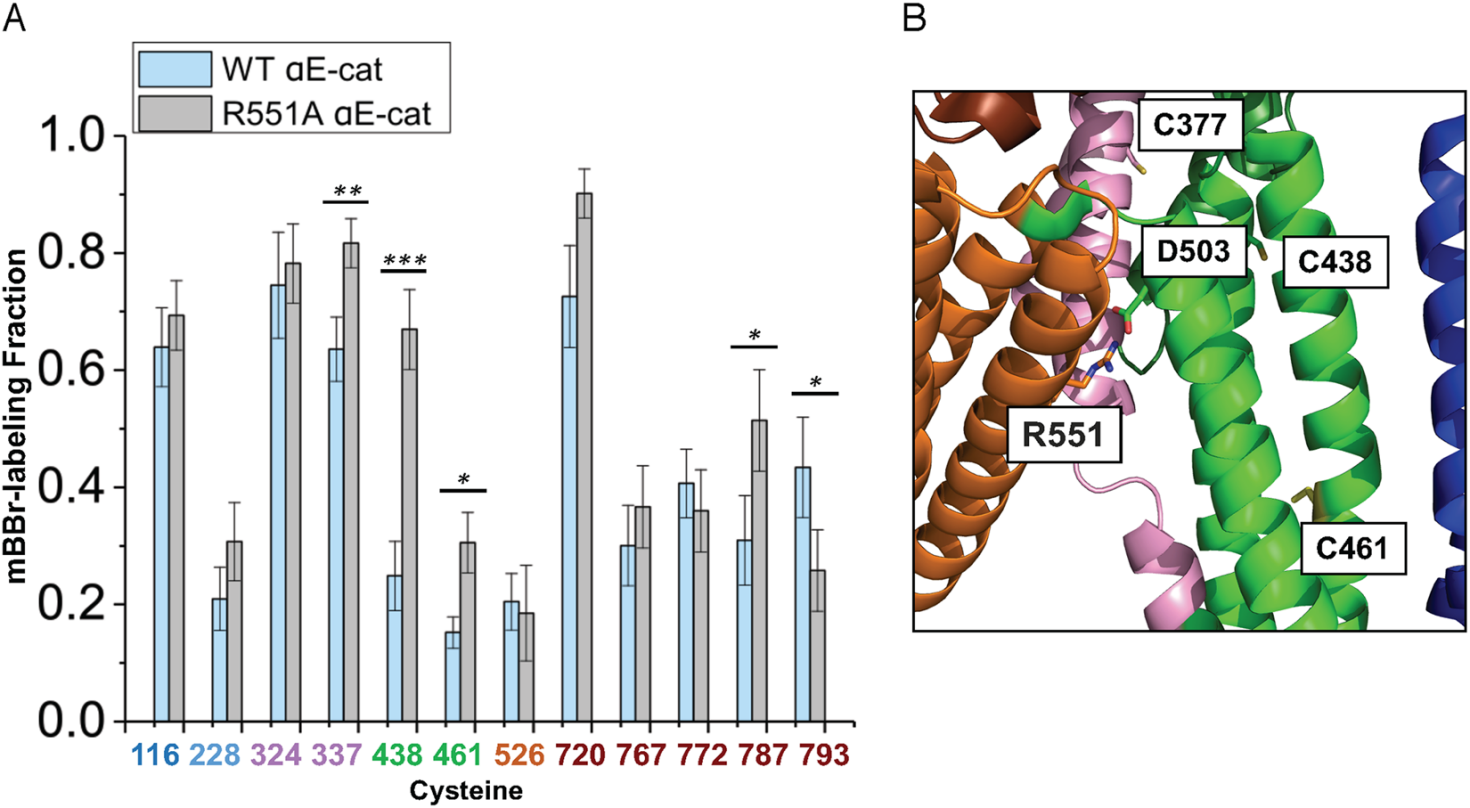
R551A αE-catenin. (**A**) Cysteine labeling of WT and R551A mutant αE-catenin by mBBr. The data for WT αE-catenin is from 7 technical replicates and 5 independent protein preparations. The data for the R551A mutant are from 4 technical replicates and 3 independent protein preparations. p-values ≤ 0.05 are flagged with one star (*), ≤0.01 with two stars (**), and ≤0.001 with three stars (***). (**B)** Proximity of C461 and the D503-R551 salt bridge in the M_II_-M_III_ interface.

**Figure 5:**
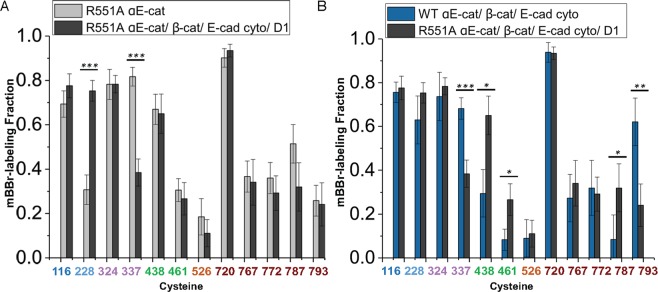
Coupling of the M domain with N and ABD indicated by cysteine labeling of R551A. (**A**). Labeling of αE-catenin R551A in the cadherin•β-catenin•R551A αE-catenin•vinculin D1 complex (8 technical replicates and three independent protein preparations), compared to that of R551A alone (see **B**). (**B**) Labeling of WT αE-catenin•cadherin•β-catenin (4 technical replicates and two independent protein preparations) and αE-catenin R551A•cadherin•β-catenin•D1complexes (same data as panel (A)). In each panel, p-values ≤ 0.05 are flagged with one star (*), ≤0.01 with two stars (**), and ≤0.001 with three stars (***).

The R551A mutation in αE-catenin disrupts the salt bridge network that links the three four-helix bundles of the M domain and thereby enhances binding to vinculin^18^. A charge reversal mutant at this site, R551E, as well as other mutations designed to disrupt the salt bridge network, likewise enhance binding to vinculin^18,30^. Using isothermal titration calorimetry (ITC), we found that full-length R551A bound to the αE-catenin binding D1 domain of vinculin with essentially the same dissociation constant as the isolated M_I_-M_II_ fragment of the wild-type αE-catenin (K_D_ = 4 nM), rather than the 475x weaker binding of the full-length wild-type protein (Table 1, Supplemental Fig. 5A). (Like wild-type αE-catenin, no binding of αE-catenin R551A to autoinhibited, full-length vinculin was observed (Table 1, Supplemental Fig. 5B)). Likewise, the ternary E-cadherin•β-catenin•αE-catenin R551A mutant complex bound strongly to vinculin D1 with a K_D_ of 26 nM (Table 1, Supplemental Fig. 5C). SAXS measurements of full-length R551A αE-catenin, as well as thermal melting data, indicated that the mutation produces no major structural changes relative to the wild-type protein nor significant stability changes in the individual helical bundles (Supplemental Fig. 4). However, increased mBBr labeling of C337 (M_I_), C438 (M_II_) and C461 (M_II_) was observed in isolated R551A vs. wild type αE-catenin (Fig. 4), consistent with increased dynamics in the mutant predicted from MD simulations of the isolated M domain^34^. We also found that addition of vinculin D1 to the E-cadherin•β-catenin•αE-catenin R551A mutant complex drastically reduced exposure of C337, whereas C324 was unaffected (Fig. 5). These latter observations further validate the significance of changes in Cys reactivity, as crystal structures of M_I_ bound to vinculin D1 revealed that C337 is buried in a hydrophobic interface with vinculin, whereas C324 lies in the connection between the two helices and is disordered in the vinculin complex^22,29,30^.

**Table 1:** Binding data for αE-catenin R551A to vinculin and β-catenin determined by ITC.

αE-catenin variant or complex	Binding molecule	K_D_ (M)	ΔH (kcal/mol)	TΔS (kcal/mol)	ΔG (kcal/mol)
αE-catenin R551A	Vinculin D1	4.1 ± 2.2 × 10^−9^	7.7 ± 1.0	19.0	−11.4
αE-catenin R551A•β-catenin•Ecyto	Vinculin D1	2.6 ± 1.4 × 10^−8^	6.7 ± 0.5	17.0	−10.3
αE-catenin R551A	Vinculin Full Length	ND	—	—	—
αE-catenin R551A	β-catenin	7.8 ± 1.7 × 10^−8^	−33.3 ± 1.8	−23.6	−9.7
αE-catenin R551A	β-catenin•Ecyto	1.4 ± 0.6 × 10^−8^	−32.3 ± 2.4	−21.6	−10.7
αE-catenin wt*	Vinculin D1	1.8 ± 0.2 × 10^−6^	12.6 ± 0.6	20.4	−7.8
αE-catenin M_I_-M_II_*	Vinculin D1	5.2 ± 0.3 × 10^−9^	9.9 ± 0.1	21.2	−11.3
αE-catenin•β-catenin•Ecyto*	Vinculin D1	1.9 ± 0.4 × 10^−6^	1.4 ± 0.1	9.2	−7.8
αE-catenin wt	Vinculin Full Length	ND	—	—	—
αE-catenin wt**	β-catenin	2.3 ± 0.4 × 10^−8^	−16.1 ± 0.7	−5.7	−10.4
αE-catenin wt**	β-catenin•Ecyto	0.9 ± 0.3 × 10^−9^	−10.1 ± 0.1	2.2	−12.3

The data shown for each binding pair are the average of three independent measurements, with the standard deviation indicated. Representative traces are shown in Supplemental Fig. 5. For comparison, the lower half of the table shows published ITC data for wild type αE-catenin complexes. ND, no binding detected.

*Ref.^22^,

**Ref.^27^.

Cysteine labeling and thermodynamic data obtained from the R551A mutant also provided direct evidence for allosteric coupling between the M domain and the flanking regions of αE-catenin. The N-terminal 56 amino acids of αE-catenin contribute to the inhibition of binding of vinculin to the M_I_ bundle^22^, indicating that the N and M domains are thermodynamically coupled. As in the wild-type case (Fig. 2), binding of the E-cadherin•β-catenin complex to R551A αE-catenin produced labeling changes at C228 in the N_II_ domain (Fig. 5A), which as noted above likely couple to the M_II_ domain. (Although the R551A data were obtained in the presence of vinculin D1, the change in C228 is not caused by vinculin binding, as its labeling is unchanged when comparing the E-cadherin•β-catenin complex with either wild-type αE-catenin or with R551A αE-catenin bound to vinculin; Fig. 5B). Given these findings, we asked whether the R551A mutation in the αE-catenin M domain would affect binding to β-catenin. Measured by ITC, the R551A mutant bound more weakly than wild type αE-catenin to both β-catenin and the E-cadherin•β-catenin complex (Table 1, Supplemental Fig. 5D,E). The R551A binding interactions are entropically very unfavorable compared to wild type αE-catenin^27^ (Table 1), suggesting the more dynamic mutant protein undergoes a greater loss of conformational entropy upon binding to β-catenin compared to the wild-type protein due to the greater intrinsic dynamics of the mutant. Taken together, the structural, thermodynamic and cysteine labeling data are consistent with a model in which N_II_-M_II_ contacts change upon binding to β-catenin and thereby couple binding of β-catenin to the rest of the molecule. Finally, we note that destabilization of the M domain by the R551A mutation leads to changes in labeling at C787 and C793 in the ABD (Fig. 4A), providing further evidence for structural communication between these domains.

### Conformational changes in αE-catenin in the presence of mechanical force

**Figure 6:**
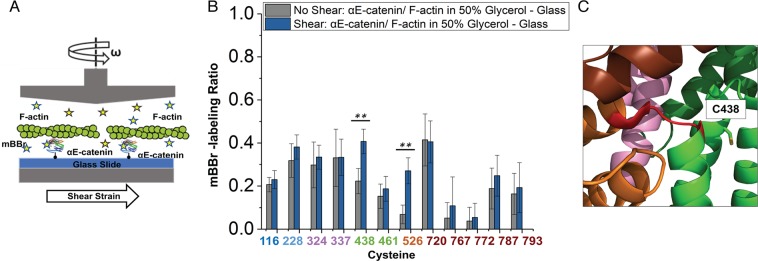
Changes in αE-catenin cysteine labeling upon shearing in the presence of F-actin. (**A**) Schematic representation of application of shear force to the αE-catenin/F-actin complex in a cone-plate rheometer. N-terminally His_8_-tagged αE-catenin is coupled to a Ni^2+^-NTA coated glass slide in the presence of F-actin, the cysteine-labeling agent (mBBr; yellow stars) and 50% glycerol. (**B**) Labeling of the αE-catenin/F-actin complex under shear. Data are from 5 technical replicates and 5 independent protein preparations. p-values ≤ 0.01 are indicated with two stars (**). (**C**) Proximity of C438 to the M_II_-M_III_ linker, shown in red.

Current high-resolution structural methods such as x-ray crystallography cannot examine molecules under tension, so we developed a method to apply force to αE-catenin in solution while simultaneously probing cysteine accessibility. We used a cone and plate rheometer^38^ to apply force to immobilized protein in tandem with site-specific labeling (Fig. 6A). In this device, a spinning cone makes a shallow angle with the underlying plate and thereby applies a uniform shear to the fluid on the plate^38^. We immobilized N-terminally His_8_-tagged αE-catenin molecules on a flat Ni^2+^-NTA-coated glass surface and subjected them to shear in the presence of mBBr and actin filaments. The proteins were then eluted with EDTA and analyzed by mass spectrometry. Unlike the labeling of pre-saturated complexes described above, it is possible that simultaneous addition of mBBr with F-actin alters the interaction of αE-catenin with F-actin. Even if so, as noted above direct comparison of sheared and unsheared samples would indicate significant changes in αE-catenin labeling and hence conformation (see below).

The force applied to the immobilized molecules can be estimated from the equation F = τ x *A*, where τ is the shear stress of the solution and *A* is the area occupied by the protein^39^. The shear stress depends upon the shear rate of the moving cone and the viscosity of the liquid solution^39^. To achieve a sufficient viscosity, 50% glycerol was present in the solution. Using a shear rate of 6,000 s^−1^, the measured viscosity was ~0.02 N s m^−2^, which is approximately 20 times that of pure water and closer to the viscosity of the cytoplasm^40^. These parameters give a shear stress τ = 120 Pa. To achieve physiologically meaningful forces, we added actin filaments at a concentration that saturates αE-catenin, which gave an average area of 0.039 μm^2^ for the αE-catenin•F-actin complex (see Experimental Procedures). With these values for the shear stress and area, we estimate that a force of ~4.7 pN is applied to αE-catenin bound to a single actin filament of average length. Complexes with longer filaments experience larger forces linearly proportional to filament length; *in vitro* polymerized filaments follow an approximately exponential distribution of lengths up to about 20 μm, so a small fraction of αE-catenin•F-actin complexes could experience forces significantly higher than 4.7 pN. Conversely, a significant fraction of filaments is shorter than 4.9 μm, with correspondingly smaller forces experienced by complexes with αE-catenin. Despite these uncertainties, given that the force regime experienced by that αE-catenin in AJs was recently determined to be 2–8 pN^12^, and that force promotes strong binding to vinculin at 4.5 pN^20,21^, the forces applied here are in a physiologically relevant range.

Before applying shear force in the cone-plate rheometer, several controls were needed. We first assessed the effect of 50% glycerol on labeling by comparing the αE-catenin•F-actin complex in buffers containing 0% and 50% glycerol (Fig. 3A). There was no consistent decrease or increase in labeling between these conditions, indicating that the higher viscosity did not affect the labeling reaction. The high glycerol concentration did, however, significantly reduce labeling at C337 (M_I_), C526 (M_III_), and C767 and C772 in the ABD. These changes might indicate some sensitivity of the dynamics of these domains to solvent properties. We next assessed whether the immobilization on glass required for the cone-and-plate rheometer measurements affects accessibility (Fig. 3B). Labeling of C116, C324 and C772 decreased between solution and immobilization on glass. It is not clear why these particular positions changed; it is possible that in the immobilized αE-catenin, these positions lie close to the glass surface and become less accessible to the probe.

Finally, we compared the labeling of glass-immobilized αE-catenin•F-actin complex in the absence and presence of shear force (Fig. 6B). Only two positions showed significant changes relative to controls: both C438 (M_II_) and C526 (M_III_) were labeled more in the presence of shear force (Fig. 6B). These changes are supported by single-molecule experiments and previously reported MD simulations^20,34^. The MD simulations indicate that the closed state is stabilized by salt bridges in the M_I_-M_III_ and M_II_-M_III_ interfaces^34^, and that mechanical perturbation causes a reorientation of the M_II_ and M_III_ domains, with formation of conformational intermediates that involves rupture of the initial and formation of new salt bridges^33^.

**Figure 7:**
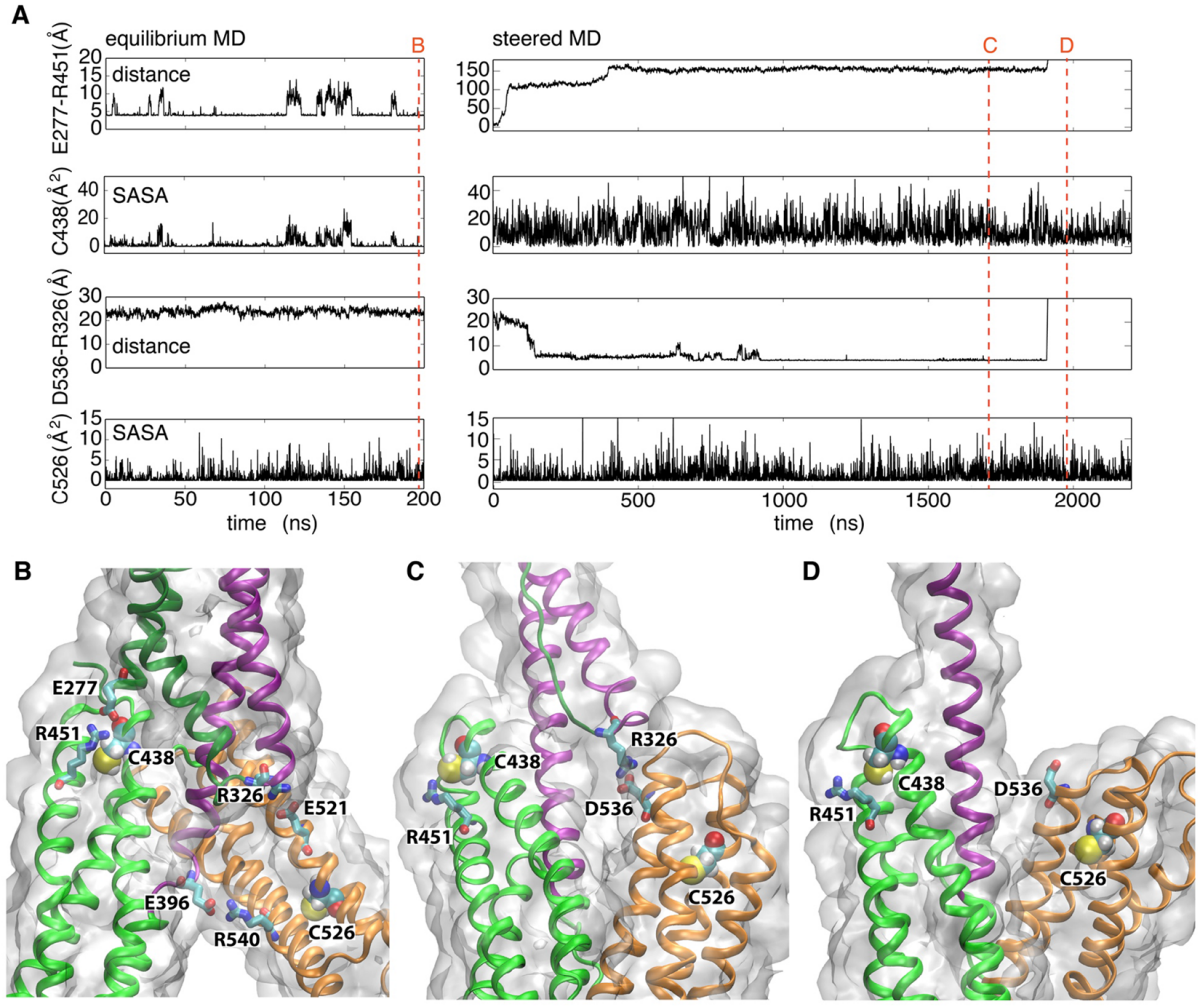
The accessibility of C438 differs in the absence and presence of external force in MD simulations. (**A**) Side chain Solvent Accessible Surface Area (SASA) of C438 and C526, and distances between ends of residues forming two relevant salt bridges, are plotted based on 200 ns equilibrium MD simulation (left) and 2.2 μs steered simulation in the presence of 100 pN external force (right). The position of snapshots shown in panels B, C, and D are highlighted as red dashed lines. (**B**–**D**) snapshots from the simulations. B is a typical snapshot from an equilibrium simulation, whereas (**C**,**D**) are snapshots before and after the rupture of R326-D536 salt bridge in the steered simulation.

To further characterize conformational changes relevant to C438 and C526, we extended our previous simulations^34^ and performed a more detailed analysis. C438 sits close to the loop connecting M_II_ to M_III_ (Fig. 6C). In the MD simulations, the change in the relative positions of these two helical bundles makes this end of M_II_ more accessible: in the absence of external force (equilibrium simulation) C438 is buried at the interface between M_I_ and M_III_ domains that is stabilized by the E277-R451 salt bridge (Fig. 7A,B), and is only transiently exposed to the surface, whereas in the presence of external force (steered simulation), the E277-R451 salt bridge is ruptured rapidly (Fig 7C,D), and accordingly the solvent accessible surface area (SASA) of C438 is increased to ~30 A^2^ (Fig. 7A).

The change in C526 is more surprising, as it is normally buried in the hydrophobic core of the M_III_ bundle (Fig. 7B). However, long-time scale simulations under force revealed that a salt bridge that forms between R326 (M_I_) and D536 (M_III_) ruptured just before separation of M_I_ from M_III_ (Fig. 7A). As C526 lies on the same helix as D536, our results may indicate that the separation of M_I_ from M_III_ enables enhanced breathing of the M_III_ bundle (Fig 7C,D). The SASA of C526 fluctuates more significantly while native M_I_/M_III_ interactions are disrupted during the steered MD simulation, compared with the equilibrium MD simulation where all the native M_I_/M_III_ interactions are maintained (Fig. 7A). The actual change in M_III_ dynamics during this process may be even more significant, but we may not be able to capture it in its entirety due to the relatively short time scale of the simulations.

Since force is associated with changes in the salt bridge network in the M domain, the apparent force-dependent coupling of the ABD and M domains under force might predict that mutations that disrupt the salt bridge network in the M domain would affect actin binding even in the absence of force. We tested the ability of αE-catenin R551A to bind actin in a co-sedimentation assay, and see no changes versus wild-type αE-catenin (S. P., N. Bax, A.D. and W.I.W., manuscript in preparation). These results indicate that force has effects beyond simply disrupting the salt bridges, likely by promoting larger conformational shifts such as whole domain movements.

## Discussion

By connecting the cadherin•β-catenin complex to F-actin, αE-catenin can sense and transmit internally generated and externally applied forces at the adherens junction. The interactions of αE-catenin are allosterically modulated by binding to its protein partners and by mechanical load. Binding of the cadherin•β-catenin complex to αE-catenin weakens association of αE-catenin with F-actin in solution, but the linkage of the cadherin•catenin complex with F-actin is strengthened by force^12^. Vinculin binds to αE-catenin weakly in solution, but removal of the M_III_ domain, also known as the “adhesion modulation domain”^41^, as well as the first 56 residues of αE-catenin, results in strong vinculin binding^22^. Force enhances binding to vinculin^20^, likely due to formation of sequential structural intermediates in the M domain that facilitate exposure of the vinculin-binding site^31,33,34^.

Given its position in the αE-catenin structure, the M domain likely serves as a transducer of allosteric communication between the N-terminal β-catenin binding domain and the ABD, but there have been few experimental data that directly probe structural changes that couple the N, M and ABDs. Here, we observed changes in cysteine exposure in the N_II_ and in M domains upon binding of αE-catenin to the E-cadherin•β-catenin complex (Figs 2B, 5A), consistent with the plasticity of the N-M interface observed in crystal structures and modeling^27^ (Fig. 2C, Supplemental Fig. 3). Due to the crosstalk between the N and M domains, binding of E-cadherin•β-catenin may regulate the interaction of the M domain with other proteins, including vinculin^22,29,30^, afadin^42^ and ZO-1^43^, and perhaps the interaction of the ABD with F-actin. In addition, we see evidence for possible allosteric coupling between the ABD and the remainder of αE-catenin, as binding with E-cadherin•β-catenin (Fig. 2B), as well as alteration of the M domain by mutation (Fig. 4A), altered labeling of the ABD. A possibility is that this coupling may occur via the N-terminal residues in αE-catenin that modulate vinculin binding^22^, but this remains to be tested.

In the shear experiments, physiologically significant forces applied to the αE-catenin•F-actin complex produced changes in the labeling of the M_II_ and M_III_ domains but not in M_I_, which is the vinculin-binding site. Although there were effects of both glycerol and glass immobilization on labeling, direct comparison of the sheared and non-sheared samples shows clear differences that are attributable to application of force. Application of force to the isolated M domain using magnetic tweezers showed that vinculin binding is promoted by 4.5–5 pN of force^20^. The absence of labeling changes at C324 and C337 (M_I_) under shear suggests that the conformational equilibrium of the M_I_ bundle does not change in response to these low-medium forces. On the other hand, the labeling of C337 changed in the R551A mutant, indicating that the mutation affects the conformational equilibrium; this was also manifest in increased labeling at C438 (Fig. 4A). This may indicate that the structural effects of force differ from those caused by removal of a single salt bridge. In any case, our findings suggest a model in which in the absence of force, M_II_ and M_III_ hinder adoption of the open vinculin-binding conformation of M_I_ and perhaps access of vinculin to the M_I_ helices, and that force relieves this inhibition by shifting their positions with respect to M_I_.

Optical trap, magnetic tweezers, and single-molecule atomic force microscopy (AFM) are well-established methods that can quantitatively apply force and detect the stress response of various mechanical anchors^44–46^, including αE-catenin^12,20,31,32^. However, these methods are not able to reveal the nature or location of force-induced structural alterations. Specific regions that undergo structural changes have been proposed by correlating force-induced molecular extensions with calculated unfolding lengths of the domains and by molecular dynamics simulations^6,19,20,31,32,34^. Here, we have developed a technique that allows direct force application to molecules in bulk, coupled with the detection of structural changes probed by changes in cysteine accessibility. Although interpreting the data from these experiments depends on prior knowledge of high-resolution structures obtained in the absence of force, it complements the biophysical and simulation data by experimentally identifying local changes upon application of force as well as the effect of complex formation with partner proteins.

## Methods

### Protein expression and purification

Murine αE-catenin constructs were cloned into a pGEX-2T vector that was modified to introduce a tobacco etch virus protease (TEV) cleavage site after the N-terminal glutathione-S-transferase (GST) affinity tag. Point mutations were introduced by site directed mutagenesis. For crystallography, the full-length construct was truncated to residues 82–883, and Cys116 of the full-length sequence was mutated to serine. The constructs were expressed in *Escherichia coli* BL21 cells. Cells were grown to OD_600_ = 0.8 at 37 °C and induced overnight at 18 °C with 0.5 mM isopropyl 1-thio-β-D-galactopyranoside. The proteins were purified on a glutathione–agarose GST affinity column. After equilibration into 20 mM Tris pH 8.0, 150 mM NaCl, 1 mM DTT, 1 mM EDTA, and 10% glycerol αE-catenin was cleaved from the GST-tag on the column by overnight incubation with TEV at 4 °C. The eluate from the GST column was purified by anion exchange chromatography (Mono Q 10/100, GE Healthcare) in 20 mM Tris pH 8.0, 1 mM DTT with a 0 to 500 mM NaCl gradient.

Following anion exchange chromatography, αE-catenin constructs were further purified by size exclusion chromatography (SEC; Hiload 16/600 Superdex S200, GE Healthcare) in different buffers depending on the experiment. For crystallization, the protein was purified in 20 mM Tris pH 8.0, 150 mM NaCl and 1 mM DTT, and concentrated to 10–20 mg ml^−1^. For ITC experiments, all proteins were prepared with 20 mM HEPES, pH 8.0, 150 mM NaCl and 1 mM DTT. This same buffer was used for the SAXS experiments on αE-catenin 82–883. To prepare αE-catenin for SEC-SAXS analysis, the size exclusion column was run in phosphate buffered saline, 1 mM DTT and 1% glycerol; pooled monomer fractions were concentrated shortly before data collection to 100 μM. Murine β-catenin, chicken vinculin, vinculin D1 domain (residues 1–259) and the cytoplasmic domain of E-cadherin were purified as described^22^.

For cysteine labeling experiments, wild-type and R551A αE-catenin were expressed using the pGEX-2T vector described above but modified to contain an 8-His tag following the TEV cleavage site. These were expressed in *Escherichia coli* BL21 cells and purified as described previously^28,47^. Monomeric αE-catenin fractions from preparative size exclusion chromatography were used immediately after purification for the solution labeling experiments and for surface immobilization. G-actin was purified from rabbit muscle acetone powder (Pel-Freez, AR)^48^, flash frozen and stored at −80 °C in G-actin buffer 5 mM Tris-HCl pH 8.0, 0.2 mM CaCl_2_, and 0.2 mM ATP). Thawed 20 μM G-actin was polymerized by addition of 10x F-actin buffer (100 mM Tris pH 7.5, 500 mM KCl, 20 mM MgCl_2_, and 10 mM ATP), followed by a 1 hour incubation at room temperature in the presence of 22 μM phalloidin (MilliporeSigma, MA).

### Thiol labeling

Ternary E-cadherin•β-catenin•αE-catenin complex, and E-cadherin•β-catenin•αE-catenin R551A mutant complex bound to D1 were formed by mixing the purified components and then purified by size exclusion chromatography (SEC; S200, GE Healthcare); for actin complexes, αE-catenin was mixed with saturating amounts of F-actin. All labeling of the proteins in solution was performed after complex formation, except for the experiments with immobilized αE-catenin on the glass slides, where the mBBr was introduced simultaneously with F-actin filaments. Cysteine labeling was achieved by a 20-minute incubation at 4 °C in the presence of 1.5 mM mBBr (ThermoFisher Scientific) in thoroughly degassed buffer containing 20 mM Hepes pH 7.5, 150 mM NaCl. For experiments without F-actin, 5 μM αE-catenin (alone or in complexes) was used. In the experiments with F-actin we employed αE-catenin at 2 μM with F-actin at 20 μM; this concentration insured saturation of αE-catenin given the estimated K_D_ of 1 μM^49^. Labeling was stopped by adding reduced glutathione to a final concentration of 5 mM for 1 hour at 4 °C. The labeled protein or protein complexes were subjected to non-reducing SDS-polyacrylamide gel electrophoresis. αE-catenin bands were excised from the gel and submitted for mass spectrometry analysis at the Stanford Mass Spectrometry Facility.

### Mass spectrometry analysis

Samples of αE-catenin excised from SDS gels were digested to peptides using trypsin/LysC (Promega, WI). No reduction or alkylation of cysteines was performed. The resulting peptides were analyzed using Liquid Chromatography Mass Spectrometry (LC-MS). In LCMS experiments, peptides were injected onto a Nanoacquity Ultra Performance Liquid Chromatography (UPLC) (Waters, MA) column and eluted at 450 nl min^−1^ flow rates, with a 0.2% aqueous formic acid Phase A and 0.2% formic acid in acetonitrile for Phase B. The analytical column was an in-house hand packed C18 reverse phase column of ~15 cm in length using 2.4 μm particles with an I.D. of 100 μm. Peptide samples were ionized by a collision-induced dissociation method and further analyzed by an Orbitrap Elite mass spectrometer (Thermo Scientific, USA). Resulted RAW files contained LC-MS/MS peptide fragmentation data. Further analysis was accomplished by Byonic version 2.14.27 (Protein Metrics) of a search algorithm that used a targeted Fasta database for αE-catenin and allowed detection of the mBBr modification of cysteines. All data were filtered and presented at a 1% false discovery rate as described previously^50^. Additional post processing to determine the labeling efficiency was completed using custom scripts in MatLab to align peptides and to determine the ratio of spectral counts observed with and without the mBBr modification. To quantify the labeling ratio of the cysteines in αE-catenin, we used a total spectral counts (SC) approach^51^. In the SC method, the labeling efficiency for a given cysteine was determined by a ratio of the total number of mBBr-modified peptides over the total (mBBr-modified and non-modified) number of peptides containing this cysteine. We note that no labeling of lysine residues, which can react slowly with mBBr, was detected.

The labeling efficiency or labeling ratio for each cysteine was calculated as a weighted mean from a series of independent experiments. The labeling efficiency from each replicate contributed to the weighted mean in proportion to the total number of the detected peptides that the given replicate contributed to the total number of peptides across a whole series. Statistical significance was determined by Welch’s T-test utilizing the R software package. We calculated the weighted standard error of the labeling ratio for each of the 12 cysteines. Furthermore, in SC analysis, the measurement error of the labeling efficiency for each cysteine was a function of the total number of detected peptides and was calculated as a signal-to-noise ratio (√n/n). The total error was calculated as a square average of the measurement and standard error.

### Isothermal titration calorimetry

ITC experiments were performed in a VP-ITC calorimeter (Microcal, GE Healthcare) in 20 mM HEPES pH 8.0, 150 mM NaCl and 1 mM DTT, the same buffer used in previous studies of αE-catenin binding to β-catenin and vinculin^22,27^ (in ref.^22^ the reducing agent was 1 mM TCEP rather than DTT). To avoid αE-catenin dimerization at higher concentrations, the monomer was placed in the cell at concentrations varying between 8–11 μM. The binding partner was injected at concentrations varying between 70–155 μM. Two 2 μl injections were followed by 30–34 9 μl injections. Experiments were performed at 25 °C. Data were analyzed with the Microcal Origin software. For baseline correction the heat signal at saturation was averaged and subtracted from all data points. For multiple measurements the weighted average was calculated.

### Rheological shearing of αE-catenin-F-actin complexes

To prepare the Ni^2+^-nitroloacetic acid (NTA) functionalized slides, NHS-activated 45 × 75 mm glass slides (Arrayit Corporation, CA) were first immersed in a solution of 0.01 M N-(5-amino-1-carboxylpentyl) iminodiacetic acid (NH_2_-NTA) in dimethylsulfoxide solution containing 0.07 M triethylamine for 20 hours at room temperature with stirring. The slides were then washed three times with dimethyl sulfoxide and twice with ethanol, with stirring, to remove excess unreacted reagents. The NTA-coated surfaces were then dried under a gentle stream of nitrogen and dipped in 50 mM NaOH for 5 minutes, and thoroughly rinsed with ultrapure water. The slides were dipped in 1 M NiCl_2_ aqueous solution for 1 hour, then rinsed with water and 1 mM ammonium citrate buffer pH 3.0 and finally kept in water until they were used.

Prior to immobilization of αE-catenin, the Ni^2+^-NTA glass surface was passivated with 10% pluronic F-127 (Sigma-Aldrich, MO) and 1% Bovine serum albumin (BSA) (Sigma-Aldrich, MO) for 1 minute and 5 minutes, respectively, with a phosphate-buffered saline (PBS) wash after each of these steps. Monomeric αE-catenin bearing an N-terminal 8-His tag at 5 μM concentration was then pipetted onto a circular area defined by a 2 cm diameter silicone O-ring (McMaster-Carr, CA) and incubated for 1-hour at 4 °C. Three washes of the glass slide with 10% Tween-20 in PBS were performed to remove unbound αE-catenin. The slides were then washed once with PBS and then stored overnight at 4 °C in F-buffer (10 mM Tris pH 7.5, 50 mM KCl, 2 mM MgCl_2_, and 1 mM ATP). Prior to rheometry experiments, the slides were washed again in fresh F-buffer.

A rheometer with cone-plate geometry^38^ was used to create uniform shear on the immobilized αE-catenin. Simultaneous cysteine labeling of unsheared and sheared αE-catenin was performed by rotation of the rheometer cone at 0 s^−1^ or 6,000 s^−1^ shear rates, respectively, in the presence of 1.5 mM mBBr, 20 μM F-actin, and 50% glycerol in F-actin buffer for 20 minutes at 16 °C. Each experiment used 10 slides in order to obtain sufficient protein for subsequent analysis. The reaction was stopped by adding 5 mM reduced glutathione in PBS and incubating for 1 hour at 4 °C. The protein was then eluted from the glass slides with 5% SDS and 10 mM EDTA, combined, and subjected to SDS-polyacrylamide gel electrophoresis. Extracted αE-catenin (in the form of gel bands) was submitted for further mass spectrometric analysis at the Stanford Mass Spectrometry Facility.

### Force calculations

In a rheometer with a cone-plate geometry, a fluid held between a moving cone of a small angle and a flat surface plate experiences a uniform shear across the radius of the cone^38^ (Fig. 6A). The shear stress *τ* at the surface of the rheometer plate is related to the shear rate, $\dot{\gamma }$, and viscosity of the material, η, by the formula: $\tau ={\rm{\eta }}\ast \dot{\gamma }$ (ref.^39^). The shear rate $\dot{\gamma }$ depends only on the angular velocity, ω, of the moving cone and the angle that it makes with the surface^39^. The viscosity of an aqueous solution rises exponentially with increasing glycerol concentration, so glycerol was used to produce physiologically relevant forces.

A glass slide bearing immobilized αE-catenin in a 2 cm diameter circle was affixed to the stationary plate of a cone-plate DHR-3 hybrid rheometer (TA Instruments, DE), so that the glass slide surface became the surface of the rheometer plate. We introduced the labeling solution (1.5 mM mBBr, 20 μM F-actin, and 50–55% glycerol in F-actin buffer), which was held by surface tension within the circle. The labeling solution is not viscoelastic and behaves as a Newtonian fluid, and every protein molecule immobilized on the surface experiences the same shear. The measured viscosity of the labeling solution was 0.02 N s m^−2^ and the shear rate was 6000 s^−1^, which gave a shear stress of 120 Pa.

To calculate the forces experienced by the complex, we first note that αE-catenin alone has a solution radius of 4.4 nm^35^, corresponding to an area too small to experience significant forces under the conditions of our experiments. However, attachment of an actin filament will greatly increase the area of the αE-catenin•F-actin complex, and thus the forces imparted by fluid shear. Actin rods attached at a single end are the most likely to have a finite tilt angle relative to the surface, which would complicate calculation of the area subjected to shear. Attachment of an αE-catenin molecule elsewhere in the rod would lead to a smaller angle between the rod and the surface, and if a single rod is attached to multiple complexes it should lie flat on the surface. To assess the effect of the extreme case of an end-on attachment of a rod with a “free hinge”, we calculated the Weissenberg number of the actin rods (the ratio of the shear rate to the rotational diffusion coefficient of the rods)^52^, using the observation that purified G actin polymerized in F-buffer with phalloidin yields actin filaments with an average length of 4.9 μm^53^, and that the width of an actin filament is approximately 8 nm. With a shear rate of 6,000 s^−1^, the Weissenberg number is 6.5 × 10^5^. Previous measurements on tethered DNA chains subjected to shear flow revealed tilt angles of less than a degree for Weissenberg numbers several orders of magnitude lower than were generated in the present experiments^54^. Thus, it is expected that the actin rods attached to αE-catenin in the presence of the shear rates employed here will essentially lie flat on the surface. We calculate the area of a filament as a rectangle, and the dimensions above give an area of 0.039 μm^2^. Using this area and the shear stress of 120 Pa, the force calculated from F = τ x *A* is 4.7 pN.

### Molecular dynamics simulations

The M domain model from chain A of the published αE-catenin structure (PDB ID 4IGG)^26^, residues 273–635, was used in the simulations. The simulations were performed as previously described^33,34^, except that the equilibrium simulations were extended to 200 ns and the steered MD (SMD) simulation^55,56^ was performed for 2.2 μs in the presence of external force (100 pN).

## Supplementary information

Supplementary Material
