# Supplementary material for: Binding partner- and force-promoted changes in αE-catenin conformation probed by native cysteine labeling
Source: Sci Rep. 2019 Oct 25;9:15375. doi: 10.1038/s41598-019-51816-3 (PMC6814714; doi:10.1038/s41598-019-51816-3)

Binding partner- and force-promoted changes in αE-catenin conformation probed by native cysteine labeling

Ksenia Terekhova, Sabine Pokutta, Yee S. Kee, Jing Li, Emad Tajkhorshid, Gerald Fuller, Alexander R. Dunn and William I. Weis

Supplemental Information

Supplemental Text

αE-catenin is a flexible molecule in solution

Two crystal structures of full-length or nearly full-length αE-catenin homodimer have been reported. Ishiyama *et al.* described a 6.5 Å structure of full-length αE-catenin in which the ABDs were disordered (PDB 4K1N)[1]. Rangarajan and Izard described the structure of the αE-catenin 82-906 dimer at 3.7 Å (PDB 4IGG), in which the ABDs in each protomer were ordered, but adopted different positions with respect to the rest of the molecule[2]. The absence of the N-terminal 81 residues, as well as the fact that the crystals were dehydrated to improve resolution of the 4IGG structure, raises the possibility that the positions of the ABDs do not represent the solution structure of the αE-catenin dimer. We determined the structure of the αE-catenin 82-883 dimer at 4.0 Å resolution without dehydration. (Residues 884-906 are not required for actin binding[3], and residues 861-906 and 878-906 were disordered in the two chains of the 4IGG crystal structure.) Although the crystallization conditions were different, αE-catenin 82-883 crystallized in the same space group and very similar unit cell dimensions as the published αE-catenin structures, and likewise contains the αE-catenin dimer in the asymmetric unit (Table 1). The overall structure of the N and M domains is very similar to the others, and the ABD is not ordered (Fig. 2A, B); the presence of the ABD was confirmed by dissolving crystals and analyzing them on SDS-PAGE (data not shown). Interestingly, the conformation of the αE-catenin 82-883 and the 4K1N structures would allow the intramolecular packing of one ABD as seen in 4IGG but would be less favorable for the second ABD due to minor steric clashes (Fig. 2B). Superposition of the structure solved here with the others results in root-mean-square deviations (RMSDs) of 2.4 Å and 1.4 Å for 4IGG and 4K1N, respectively, whereas the individual protomers superimpose with much smaller RMSDs ranging from 0.8 - 1.2 Å.

In order to compare solution structural data to the 4IGG crystal structure, we measured small angle x-ray scattering (SAXS) data from the 82-883 dimer construct. Comparison with the scattering curve calculated from the crystal structure shows very poor agreement (Fig. 2C). The radius of gyration (shape R_g) calculated from the crystal structure is 39 Å, much smaller than the measured R_g of 56 Å and the distance distribution function P(r) derived from the SAXS data shows a more extended structure (Fig. 2D). Similar findings were reported in SAXS studies of full-length αE-catenin[1,4]. The SAXS measurements and the lack of electron density for the ABD in the present crystal structure and 4K1N[1] indicate that full-length αE-catenin contains a flexibly linked ABD and does not form a compact structure in solution. Whether the compact structure with ordered ABDs visualized in the 4IGG structure represents a minor population of the solution ensemble remains to be determined; evidence presented below is consistent with communication between

the ABD and the N-M region of αE-catenin. Conformational heterogeneity of the M_I bundle (see also below) may also contribute to the larger R_g compared to the crystal structure; previously published modeling of αE-catenin SAXS data treated the M domain as a rigid unit as observed in the crystal structures[4].

Supplemental Methods

Small Angle X-ray Scattering (SAXS)

SAXS data were collected at beamline 4-2 at the Stanford Synchrotron Radiation Lightsource. Scattering data for αE-catenin 82-883 were obtained at 5 different concentrations (10, 5, 2.5, 1.25, and 0.6 mg ml^{-1}) in 20 mM HEPES, pH 8.0, 150 mM NaCl and 1mM DTT buffer. 1 s exposures were recorded on a Pilatus 3XIM detector with a 0.1 x 0.1 mm beam and a 655 mm sample - detector distance. Scattering curves from 5 concentrations were merged and the radius of gyration R_g was determined by Guinier analysis using autoR_g in the program Primus[5]. The P(r) function was calculated with GNOM[6] using an s range extended to 0.3 $Å^{-1}$.

To obtain data from monodisperse samples of full-length wild type and R551A αE-catenin monomer and dimer, the SEC-coupled SAXS setup at the beamline was utilized. 100 µl of 10 mg ml^{-1} wild type or R551A αE-catenin were injected onto a small-volume size exclusion column (Superdex PC3.2/200) equilibrated with PBS, 1mM DTT and 1% glycerol at a flow rate of 0.05 ml/min. The column eluate was directed through a quartz capillary and was exposed to the beam in 1 s intervals. Data were recorded on a Pilatus 3XIM detector with a 0.3 x 0.3 mm beam and a 1.7 m sample–detector distance, and processed with the program SasTool. The first 100 images were averaged and used as a buffer profile, which was then subtracted from the subsequent images. R_g is automatically calculated for each frame and then plotted together with the extrapolated I(0) value. Inspection of frames across the elution peak allows to select and average datasets with minimal contamination of either monomer or dimer. Frames 380-399 and 380-389 were averaged for wild-type and R551A monomer and frames 290-299 were averaged for wild-type and R551A dimer to determine the R_g and the P(r) function. The programs Primus and GNOM were used for analysis. The R_g was determined from the linear region of the Guinier plot with an sR_g limit < 1.3. The program Crysol[7] was used to compute the scattering curve of the αE-catenin crystal structure (PDB 4IGG).

Crystallographic analysis of αE-catenin 82-883

Hexagonal crystals of αE-catenin 82-883 were grown at 22 °C by hanging drop vapor diffusion with a well solution containing 50 mM Tris, pH 8.5, 60 mM $LiSO_4$, 23.5 % PEG 400. Typical crystal dimensions were 100 x 100 x 100 $µm^3$. Crystals were frozen in liquid nitrogen without additional cryoprotectant. Data were collected in 0.5° rotation frames on a Pilatus 6M detector at BL 11-1 of the Stanford Synchrotron Radiation Laboratory. Data were integrated using XDS[8] and scaled using the program Aimless in the CCP4 program package[9]. The data were anisotropic, extending to 4.0 Å in the strongest (*hk* plane) direction, and 4.5 Å in the weaker (*l* axis) direction, as assessed by $CC_{1/2}$ > 0.5 (Ref. [9]). Statistics are shown in Table S1.

The structure of αE-catenin 82-883 was solved by molecular replacement using copy A of the αE-catenin 82-906 dimer structure (PDB code: 4IGG) as a search model. Two copies of the model could be placed using the program Phaser[10]. Model refinement was carried out with the program Phenix[11]. The first round of rigid body refinement allowing the two copies to move individually resulted in R_{work} = 45.8% and R_{free} = 46.5%. After several rounds of rigid body refinement where the molecule was broken up into individual domains the R-factors dropped to R_{work} = 31.0% and R_{free} = 34.6%. Further refinement of the structure used interactive cycles of manual building with positional and grouped temperature factor refinement with secondary structure restraints. The refined model statistics are given in Table S1, and a representative portion of the electron density map is shown in Supplemental Figure 6. The coordinates and structure factors for αE-catenin (83-882) are available from the Protein Data Bank under accession code 6O3E.

Circular Dichroism measurements

CD melting curves of wild-type and R551A mutant αE-catenin monomer were recorded with an Aviv 202-01 (AVIV Biomedical, Inc.) or a Jasco J-815 (Jasco Analytical Instruments) CD spectrometer. Measurements were performed at 2.5 μM concentration in phosphate buffered saline with 1 mM DTT. CD melting curves were measured from 10 – 95 °C at 222 nm at 1 °C intervals. For comparison of wild-type and R551A mutant melting curves, the CD signal was normalized. Melting curves were fitted to a two-state unfolding model[12] using an analysis program written in Python.

Supplemental References

1 Ishiyama, N. *et al.* An autoinhibited structure of alpha-catenin and its implications for vinculin recruitment to adherens junctions. *J Biol Chem* **288**, 15913-15925, doi:10.1074/jbc.M113.453928 (2013).

2 Rangarajan, E. S. & Izard, T. Dimer asymmetry defines alpha-catenin interactions. *Nat Struct Mol Biol* **20**, 188-193, doi:10.1038/nsmb.2479 (2013).

3 Pappas, D. J. & Rimm, D. L. Direct interaction of the C-terminal domain of α-catenin and F-actin is necessary for stabilized cell-cell adhesion. *Cell Commun Adhesion* **13**, 151-170 (2006).

4 Nicholl, I. D. *et al.* alpha-Catenin Structure and Nanoscale Dynamics in Solution and in Complex with F-Actin. *Biophys J* **115**, 642-654, doi:10.1016/j.bpj.2018.07.005 (2018).

5 Konarev, P. V., Volkov, V. V., Sokolova, A. V., Koch, M. H. J. & Svergun, D. I. PRIMUS: a windows PC-based system for small-angle scattering data analysis. *J Appl Crystallogr* **36**, 1277-1282 (2003).

6 Semenyuk, A. V. & Svergun, D. I. GNOM - a program package for small-angle scattering data processing. *J. Appl. Crystallogr.* **24**, 537-540 (1991).

7 Svergun, D., Barberato, C. & Koch, M. H. J. CRYSOL: a program to evaluate x-ray solution scattering of biological macromolecules from atomic coordinates. *J. Appl. Cryst.* **28**, 768-773 (1995).

8 Kabsch, W. XDS. *Acta Crystallogr* **D66**, 125-132 (2010).

9 Winn, M. D. *et al.* Overview of the CCP4 suite and current developments. *Acta crystallographica. Section D, Biological crystallography* **67**, 235-242, doi:10.1107/S0907444910045749 (2011).

10 McCoy, A. J. *et al.* Phaser crystallographic software. *J Appl Crystallogr* **40**, 658-674, doi:10.1107/S0021889807021206 (2007).

11 Adams, P. D. *et al.* PHENIX: a comprehensive Python-based system for macromolecular structure solution. *Acta crystallographica. Section D, Biological crystallography* **66**, 213-221, doi:10.1107/S0907444909052925 (2010).

12 Greenfield, N. J. Using circular dichroism collected as a function of temperature to determine the thermodynamics of protein unfolding and binding interactions. *Nat Protoc* **1**, 2527-2535 (2006).

Supplemental Table 1. Crystallographic data for αE-catenin 82-883.

Data collection	
Wavelength (Å)	0.9795
Space group	$P3_2$
Unit cell parameters *a, b*, *c* (Å)	145.3, 145.3, 136.3
Resolution (Å) (last shell)	20.0-4.00 (4.28-4.00)
mean I/σ(I)	7.6 (1.1)
Completeness (%)	97.6% (98.8%)
Multiplicity	3.5 (3.5)
R_{merge}	0.080 (1.240)
CC½	0.998 (0.563)
Model refinement	
No. of reflections working set (test set)	25,046 (1324)
R_{cryst}/R_{free}	0.21/0.24
Bond length r.m.s.d. from ideal (Å)	0.003
Bond angle r.m.s.d. from ideal (°)	0.554
Ramachandran analysis	
Favored regions (%)	95.7
Allowed regions (%)	4.3
Outliers (%)	0

Supplemental Figure Legends

Supplemental Figure 1. Structure of αE-catenin 82-883. A) Superposition of the αE-catenin 82-883 (colored as in Fig. 1) with that of 4KN1[1] (grey). **B)** Superposition of αE-catenin 82-883 (colored as in Fig. 1) and 4IGG[2] (grey). The N_I-domain (84-149) of the dimer structures were aligned; only one protomer (chain B) is shown. The black arrow indicates steric clashes between the actin-binding domain visible in 4IGG and αE-catenin 82-883 that would occur between the top of the ABD five-helix bundle and helices 3 and 4 of the M_{III} domain in αE-catenin 82-883. **C)** Comparison of the measured SAXS scattering curve of αE-catenin 82-883 dimer (blue) and the calculated scattering curve of the αE-catenin crystal structure (red) (4IGG). The Guinier plot is shown in the insert. **D)** Comparison of the P(r) functions of αE-catenin 82-883 obtained from the SAXS data (blue) and calculated from the αE-catenin 82-906 crystal structure (4IGG) (red).

Supplemental Figure 2. Labeling of αE-catenin. The labeling of 15 μM αE-catenin with 1.5 mM mBBr is complete after 20 min, consistent with the rapid chemistry of thiol modification. Aliquots at each time point were passed over a desalting column to remove unreacted mBBr, then measured for protein concentration by absorption at 280 nm (grey bars) and mBBr labeling by absorption at 398 nm (blue bars). The data are from three replicates of the labeling reaction from one protein preparation.

Supplemental Figure 3. Different N_{II}-M_{II} interfaces in the two protomers of αE-catenin 82-883. Detailed side chain interactions (salt bridges and hydrogen bonds) between N_{II} and M_{II} in protomers A and B. Side chains involved in hydrogen boding or salt bridge formation seen in both conformations are shown in *cyan*. Side chain interactions that are specific to one conformation are shown in *yellow* (copy A) or *orange* (copy B).

Supplemental Figure 4. SAXS analysis of WT and R551A αE-catenin. A) Scattering curves of WT (*red*) and R551A (*blue*) αE-catenin monomers. Guinier plots and the calculated radius of gyration R_g are shown below the scattering curves. **B)** Scattering curves of WT (*red*) and R551A (*blue*) dimers. Guinier plots and the calculated radius of gyration R_g are shown below the scattering curves. dimer. **C, D)** SEC-SAXS elution profile of WT (C) and R551A (D) αE-catenin. **E)** Distance distribution function of WT (*red*) and R551A (*blue*) αE-catenin monomers (solid line) and dimers (dashed line). **F)** Thermal melting curves measured by circular dichroism. WT αE-catenin is shown in *red* and the R551A mutant in *blue*. Fits assuming a two-state unfolding process are shown as solid lines; dots represent the measured data.

Supplemental Figure 5. ITC data for αE-catenin R551A binding to β-catenin or vinculin. Representative titrations are shown for the indicated binding reactions. The thermodynamic values derived from these data are shown in Table 1.

Supplemental Figure 6. Electron density for αE-catenin 82-883. A portion of the $2F_o$-F_c electron density map of αE-catenin 82-883, in the M_I region. The map is contoured at 1.0 σ and shown in wall-eye stereo.

Fig. S1

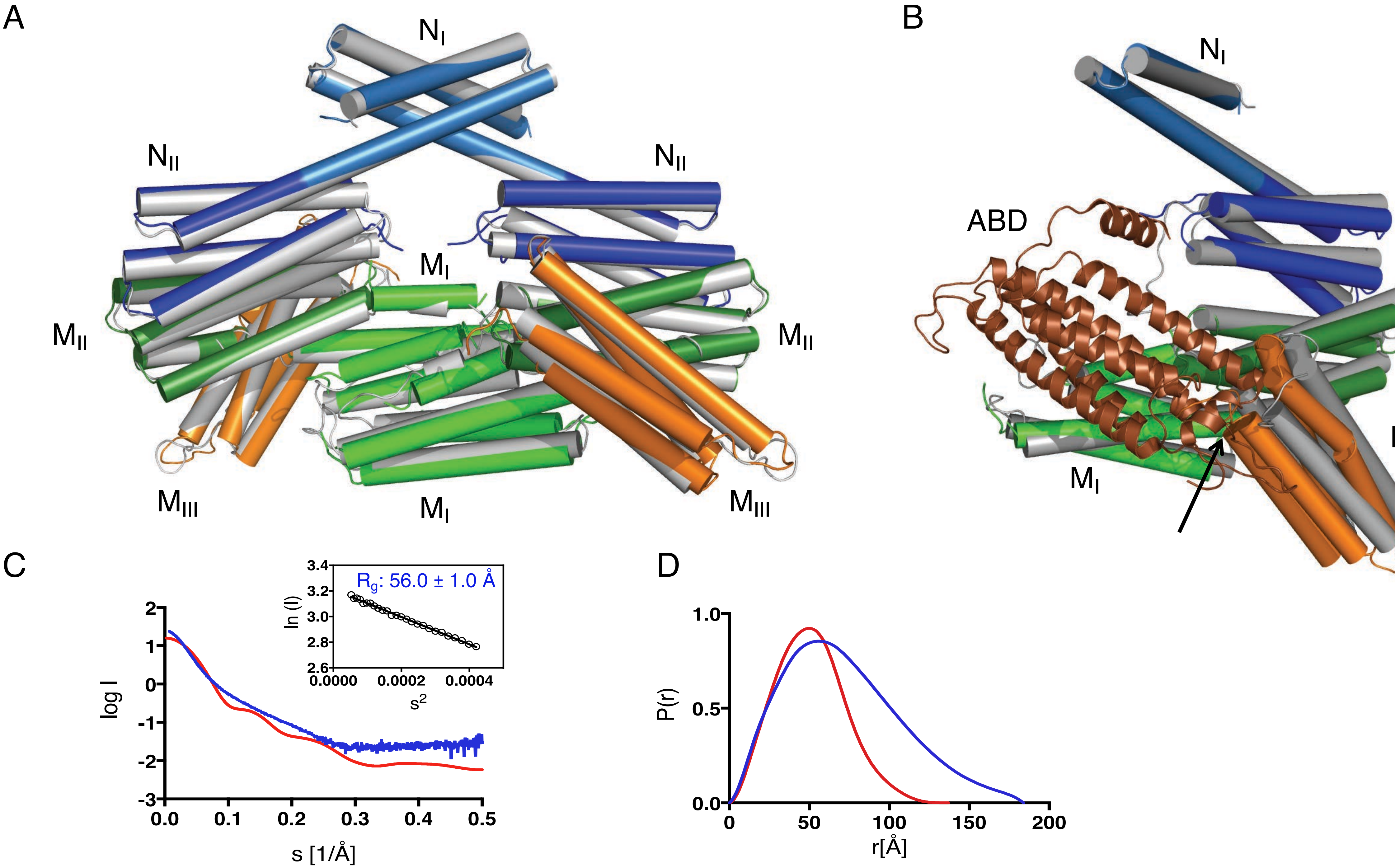

A
N_I
N_{II}
N_{II}
M_I
M_{II}
M_{II}
M_{III}
M_I
M_{III}
B
N_I
ABD
N_{II}
M_{II}
M_{III}
M_I
C
R_g: 56.0 ± 1.0 Å
ln (I)
3.4
3.2
3.0
2.8
2.6
0.0000
0.0002
0.0004
s^2
log I
2
1
0
-1
-2
-3
0.0
0.1
0.2
0.3
0.4
0.5
s [1/Å]
D
P(r)
1.0
0.5
0.0
0
50
100
150
200
r[Å]

Fig. S2

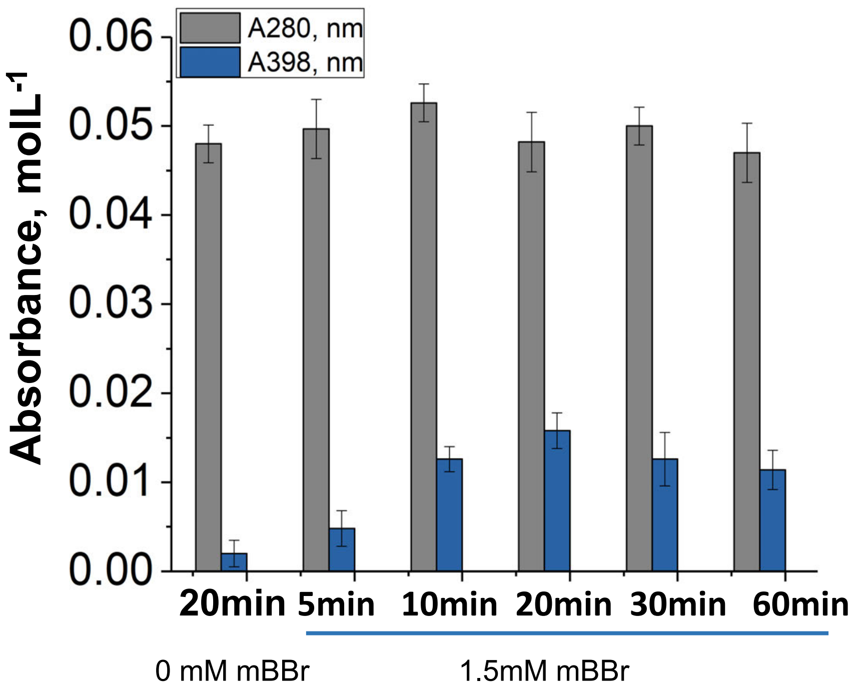
A280, nm
A398, nm
Absorbance, molL-1
0.06
0.05
0.04
0.03
0.02
0.01
0.00
20min
5min
10min
20min
30min
60min
0 mM mBBr
1.5mM mBBr

Fig. S3

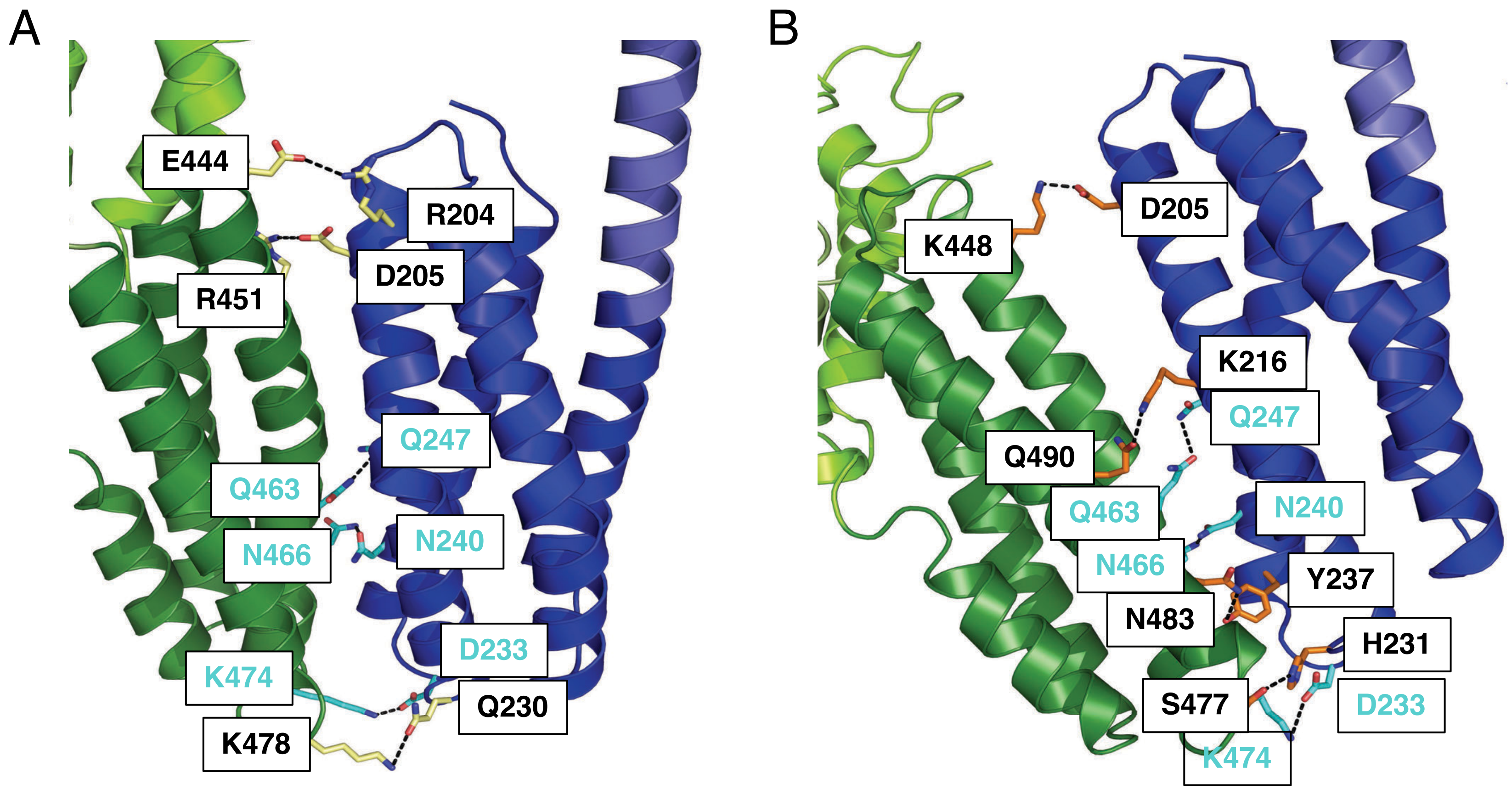
A
E444
R204
D205
R451
Q247
Q463
N466
N240
D233
K474
Q230
K478
B
D205
K448
K216
Q247
Q490
Q463
N240
N466
Y237
N483
H231
S477
D233
K474

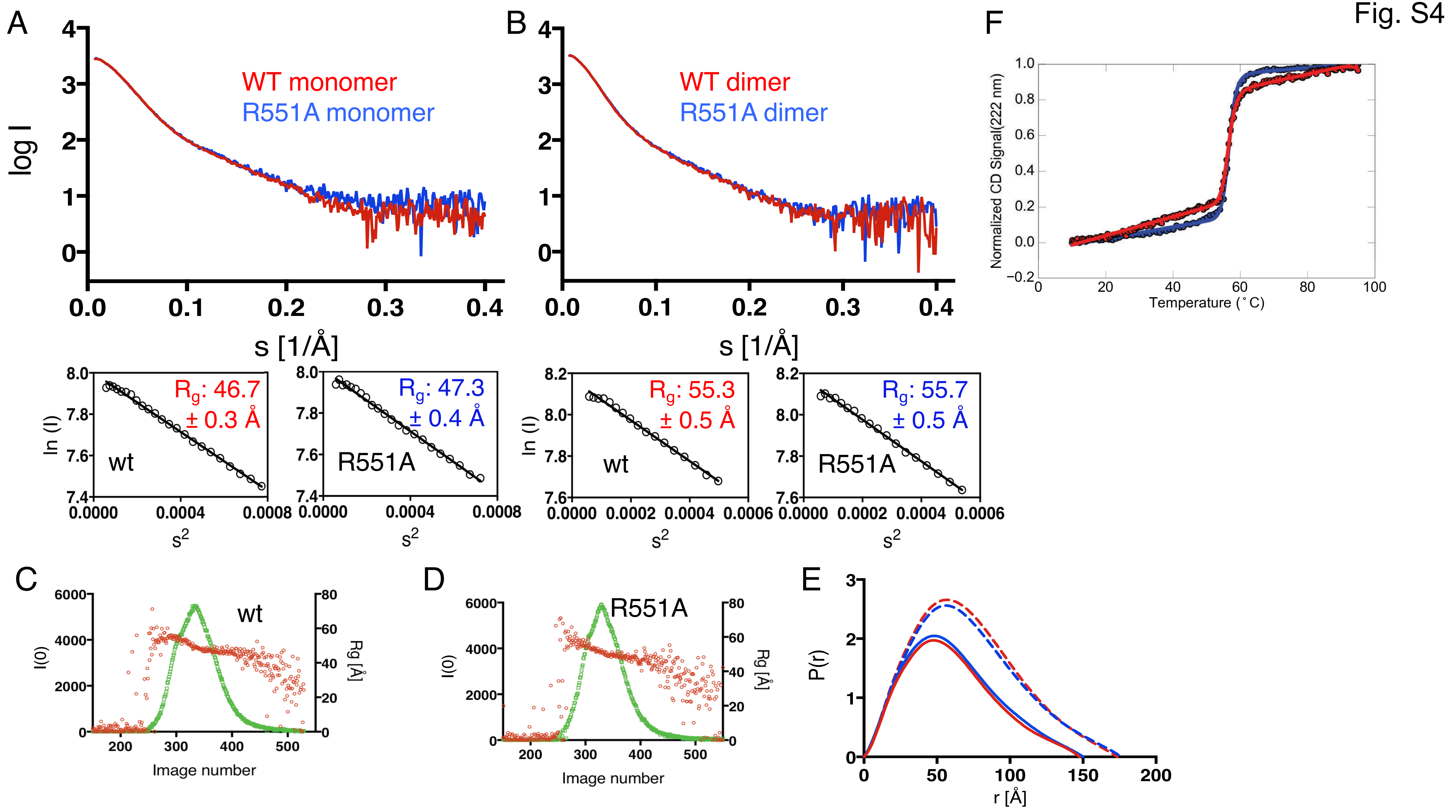
Fig. S4
A
log I
WT monomer
R551A monomer
s [1/Å]
B
WT dimer
R551A dimer
s [1/Å]
ln (I)
R_g: 46.7 ± 0.3 Å
wt
R_g: 47.3 ± 0.4 Å
R551A
R_g: 55.3 ± 0.5 Å
wt
R_g: 55.7 ± 0.5 Å
R551A
s^2
C
wt
I(0)
Rg [Å]
Image number
D
R551A
I(0)
Rg [Å]
Image number
E
P(r)
r [Å]
F
Normalized CD Signal(222 nm)
Temperature (˚C)

Fig. S5

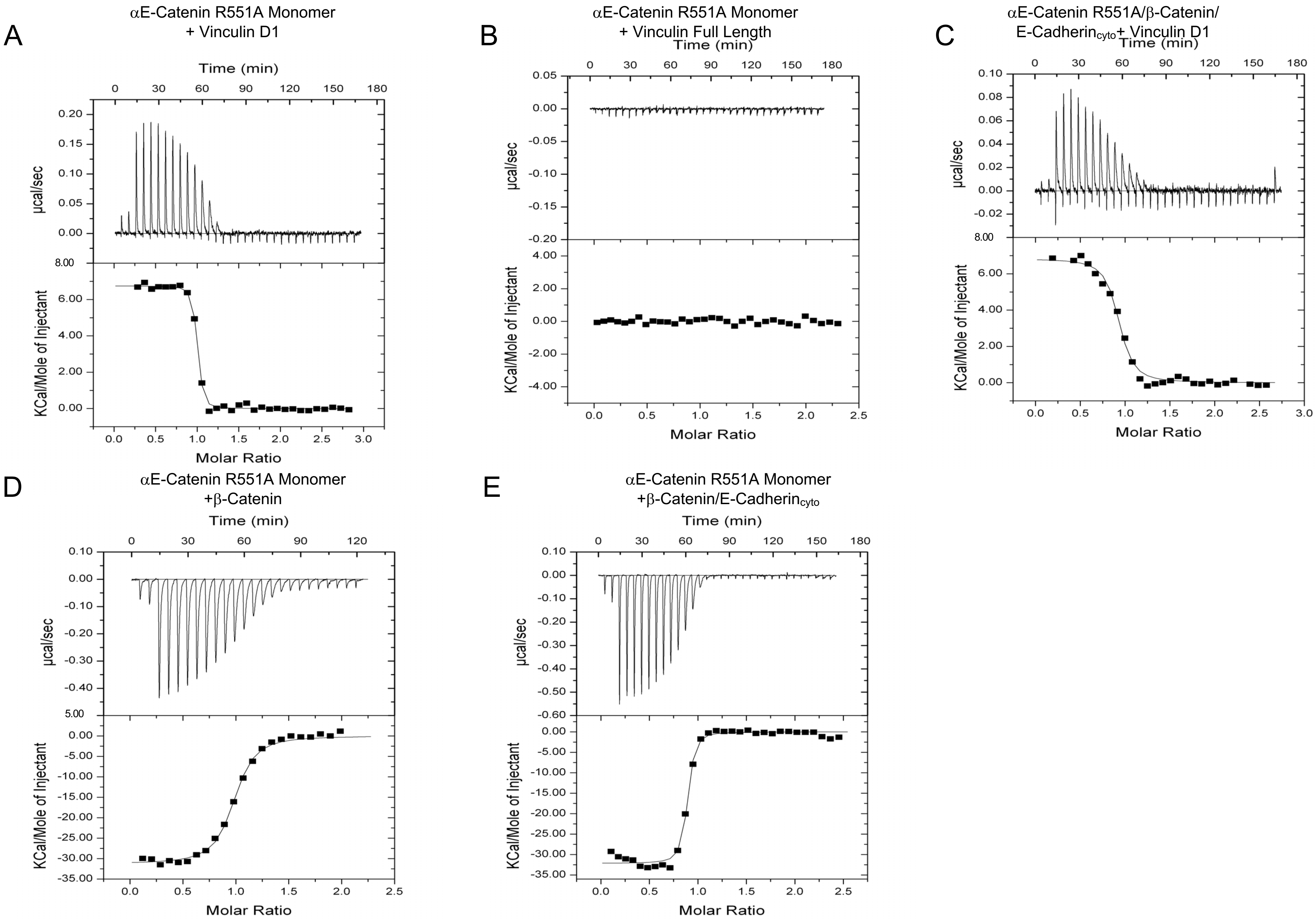
A
αE-Catenin R551A Monomer
+ Vinculin D1
B
αE-Catenin R551A Monomer
+ Vinculin Full Length
C
αE-Catenin R551A/β-Catenin/
E-Cadherin$_{cyto}$+ Vinculin D1
D
αE-Catenin R551A Monomer
+β-Catenin
E
αE-Catenin R551A Monomer
+β-Catenin/E-Cadherin$_{cyto}$
Time (min)
µcal/sec
KCal/Mole of Injectant
Molar Ratio

Fig. S6

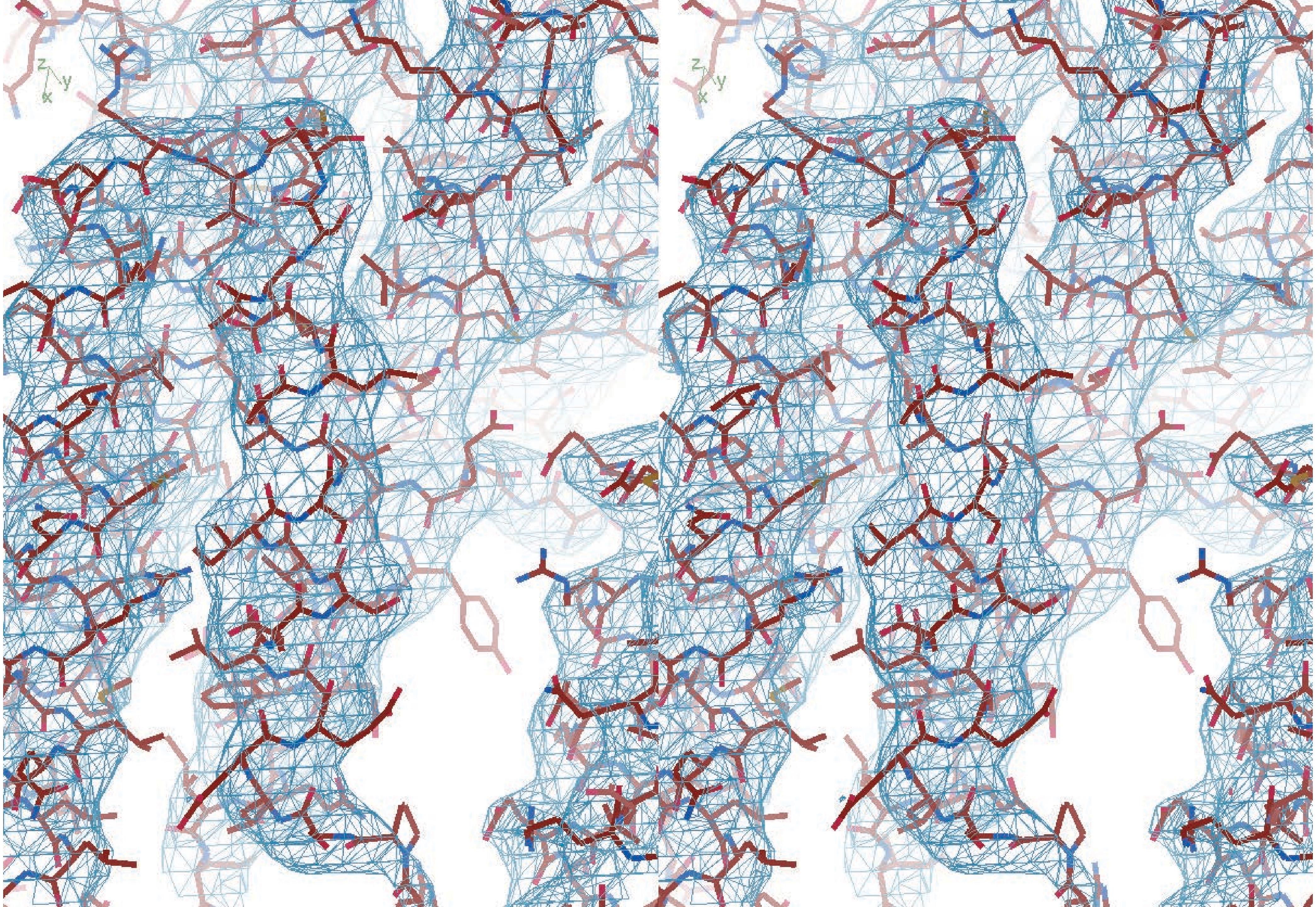

Supplement: Supplementary file 1 — Supplementary Material [file 41598_2019_51816_MOESM1_ESM.pdf]
